# Skin Recurrence of Transformed Mycosis Fungoides Postumbilical Cord Blood Transplant despite Complete Donor Chimerism

**DOI:** 10.1155/2014/743856

**Published:** 2014-12-11

**Authors:** Rahul Pawar, Anup Kasi Loknath Kumar, Janet Woodroof, Wei Cui, Joseph McGuirk, Sunil Abhyankar, Sid Ganguly, Anurag Singh, Tara Lin, Omar Aljitawi

**Affiliations:** ^1^Department of Internal Medicine, University of Kansas Medical Center, Mailstop 2027, 3901 Rainbow Boulevard, Kansas City, KS 66160, USA; ^2^Division of Hematology and Oncology, University of Kansas Medical Center, 2330 Shawnee Mission Parkway, Westwood, KS 66205, USA; ^3^Division of Pathology, University of Kansas Medical Center, 3901 Rainbow Boulevard, Kansas City, KS 66160, USA

## Abstract

*Background.* Allogeneic stem cell transplant is the treatment of choice for systemic cutaneous T-cell lymphoma (CTCL) which provides graft-versus-lymphoma effect. Herein we discuss a case of recurrence of CTCL skin lesions after cord blood transplant in a patient who continued to have 100% donor chimerism in bone marrow. *Case Presentation.* A 48-year-old female with history of mycosis fungoides (MF) presented with biopsy proven large cell transformation of MF. PET scan revealed multiple adenopathy in abdomen and chest suspicious for lymphoma and skin biopsy showed large cell transformation. She was treated with multiple cycles of chemotherapy. Posttherapy PET scan showed resolution of lymphadenopathy. Later she underwent ablative preparative regimen followed by single cord blood transplant. Bone marrow chimerism studies at day +60 after transplant showed 100% donor cells without presence of lymphoma. However 5 months after transplant she had recurrence of MF with the same genotype as prior skin lesion. Bone marrow chimerism study continued to show 100% donor cells. *Conclusion.* A differential graft-versus-lymphoma effect in our case prevented lymphoma recurrence systemically but failed to do so in skin. We hypothesize that this response may be due to presence of other factors in the bone marrow and lymph node microenvironments preventing recurrence in these sites.

## 1. Background

Cutaneous T-cell lymphoma (CTCL) is a group of lymphoproliferative disorders characterized by infiltration of malignant T lymphocytes to the skin [1–3]. WHO-EORTC (World Health Organization—European Organization for Research and Treatment of Cancer) classifies CTCL into various subgroups among which mycosis fungoides (MF) and Sézary syndrome (SS) constitute the most common forms of CTCL [1, 3–5]. Advanced and transformed MF and SS need systemic therapy. Based on current evidence allogeneic stem cell transplant is a standard treatment modality used for transformation of CTCL with a curative intent [2, 3, 5–8] and is associated with durable response [9] likely due to graft-versus-lymphoma (GVL) effect [7, 8]. Limited data exists in the form of case reports regarding effectiveness of cord blood transplant in this setting [10–12]. To our knowledge no studies have been performed comparing the efficacy of allogeneic stem cell transplantation with umbilical cord blood transplantation in the treatment of advanced CTCL. Here we report a case of a transformed MF who received myeloablative single unit umbilical cord blood (sUCB) transplantation. After biopsy proven resolution of MF with chemotherapy and attainment of 100% donor chimerism status, she experienced recurrence of MF skin lesions despite concomitant full donor chimerism in her bone marrow. Our case highlights the pathobiological aspects of this important but rare differential GVL response of extramedullary compared to intramedullary disease.

## 2. Case Presentation

A 48-year-old Caucasian female with a 10-year history of MF was referred to our bone marrow transplantation clinic for evaluation of biopsy proven large cell transformation of her MF. On presentation her only symptom was itching at sites of MF plaque-like lesions which involved her trunk and extremities in addition to tumor formation on her neck as well as left lower abdomen, right shoulder, right upper back, and right mid back. At time of referral, she was on topical treatment with UVB therapy. Her positron emission tomography (PET) scan showed multifocal lymphadenopathy in the abdomen and chest in addition to multiple nodular lesions in both lungs suspicious for lymphoma. A skin biopsy done earlier showed large cell transformation with CD4 positive T-cells which show aberrant loss of CD3 and CD7. The density and distribution of T-cells was consistent with tumor stage MF. Based on the above findings the patient was diagnosed with stage 4 MF with large cell transformation.

Systemic therapy was initiated with cyclophosphamide, adriamycin, vincristine, and prednisone (CHOP) regimen and due to limited response to first cycle of CHOP, etoposide was added to her second chemotherapy cycle. With continued limited response her chemotherapy regimen was switched to ifosfamide, carboplatin, and etoposide (ICE) as salvage therapy. Repeat PET showed improvement of abdominal and lung lesions following which she received an ablative preparative regime of fludarabine, total body irradiation (1320 cGy), and Cytoxan and sUCB (single unit umbilical cord blood) transplant. Mycophenolate was stopped at day +30 and cyclosporine was tapered following day +100. Posttransplant course was complicated by development of acute skin and gastrointestinal graft-versus-host disease (GvHD), which responded to steroids. Her day +60 peripheral blood chimerism analysis revealed 100% donor cells. Posttransplant day +100 PET scan showed complete remission of her lymphoma.

Five months after transplant she experienced recurrence of several erythematous plaque-like lesions involving her flanks, back, left neck, proximal inner right thigh, and left posterior thigh. She was off posttransplant immunosuppression at this time but was on steroids for treatment of her GvHD. A repeat biopsy of the lesion showed a mix of tumor and plaque stage MF. T-cell immunophenotype was CD4 positive and CD3/7/8 negative with scant CD30 positivity which was consistent with her pretransplant skin biopsy phenotype. Repeat chimerism analysis on bone marrow continued to show 100% donor cells. Steroids were slowly tapered off and she was started on escalating doses of interferon therapy and also bexarotene without systemic chemotherapy. Skin lesion's chimerism analysis showed 70% donor cells consistent with trafficking of donor cells to skin lesions.

She is currently 2 years after transplant and continues to have CTCL skin lesions. Her latest skin biopsy performed as a part of annual posttransplant evaluation continued to show MF; however, her bone marrow biopsy then showed 100% donor chimerism without any evidence of lymphoma confirmed either by morphology or by flow cytometry.

**Figure 1:**
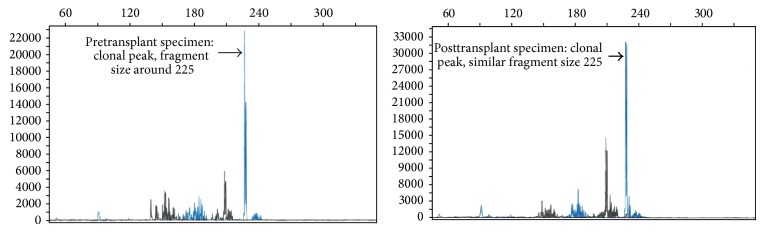
PCR for T-cell gene rearrangement in the pre- and posttransplant skin specimens revealed similar peaks, compatible with the presence of the same clone; *x*-axis shows size of restriction fragment and *y*-axis shows height of peak.

Lastly, to further investigate our case we performed T-cell gene rearrangement study on pre- and posttransplant skin specimens according to national guidelines. The study proved that lymphoma cells from posttransplant skin lesions were the same clones from pretransplant skin lesions based on the size and the region of peaks as shown in Figure 1.

## 3. Discussion

The overall prognosis in MF patients depends on the disease stage and clinical presentation, which are the key determinants of their clinical management. Early-stage MF is associated with a very good prognosis and is managed with topical therapy. Advanced and transformed MF and SS need systemic therapy. Total skin electron beam therapy (TSEBT) is an effective mean to reduce tumor burden and was used in one study [13] as a preparative regimen prior to allogeneic stem cell transplantation only in case of extensive tumor burden prior to transplantation. It was not used in our case as it is not a standard preparative regimen prior to allogeneic stem cell transplant [14–16] and because of the low tumor burden at time of transplantation. Recent studies show that allogeneic stem cell transplant is a standard treatment modality used for transformed CTCL with a curative intent [2, 3, 5–8]. Allogeneic stem cell transplant for CTCL is associated with durable response [9] likely due to GVL effect [7, 8]. However, the patient in our case developed recurrence of skin lesions despite attaining 100% bone marrow donor chimerism. The phenomenon of GVL effect which usually protects against recurrence of CTCL and which is the basis of treating CTCL cases with allogeneic transplantation appeared to provide protection against systemic nodal recurrence but seemed to fail in protecting against cutaneous recurrence [5, 17–19]. The augmentation of the GVL effect is essential not only for maintaining complete remission but also to prevent disease relapse. Donor lymphocyte infusion induced GVL effect has been shown to be effective in posttransplant relapse setting but this could not be attempted in our patient as she received cord blood transplant [19–22]. Bexarotene immunomodulation is a known approach and was attempted in our case but failed to derive any significant clinical benefit [22].

Two previous case reports describe the development of CTCL following allotransplant transmitted from allogeneic donor [23, 24]. In our case, the skin lesions were thought to be recurrence of her old CTCL lesions. This notion was supported molecularly by T-cell receptor gene rearrangement studies and by the phenotype of the skin biopsy which was CD4 positive and CD3/7/8 negative with scant CD30 positivity which was similar to pretransplant phenotype. It may also be possible that incomplete myeloablation might allow residual CTCL to survive and recur or metastasize to any part of the body. This phenomenon has been observed with relapse of SS after reduced intensity conditioning transplant despite 100% donor chimerism in the bone marrow [20]. However, our patient received complete myeloablative regimen prior to transplant and attained 100% donor chimerism in bone marrow which was negative for lymphoma; hence new occurrence of CTCL lesions in skin by migration of malignant T-cells from bone marrow was thought to be unlikely.

Our patient received steroids to combat acute GvHD. Though she was off immunosuppressant therapy at the time of recurrence, steroids which were used to treat her GvHD could have potentially contributed to recurrence of CTCL. It has been shown that negative modulation of GvHD by immunosuppressant therapy correlates with reduced GVL effect and hence recurrence of CTCL [22].

How do we explain our findings based on CTCL pathogenesis? The definitive etiology of CTCL remains to be determined; however, it has been hypothesized that continuous antigen or viral stimulation leads to formation of malignant T-cells [4]. Additionally, elevation of various chemokine levels in skin lesions leads to migration of malignant T-cells from vessels to the skin sites [4]. CTCL cells express a receptor known as cutaneous lymphocytes associated antigen (CLA), the ligand for E-selectin (CD62E). Additional adhesions molecules and skin-homing chemokines, such as C-C chemokine receptor 4 (CCR4), CCR10, and their respective ligands chemokine (C-C motif) ligand/thymus and activation-regulated chemokine (CCL17/TARC) and chemokine (C-C motif) ligand 27/cutaneous T-cell attracting chemokine (CCL27/CTACK), play a role in attracting T-cells to the skin [4, 25]. However, the cascade of events that leads to the margination and extravasation of T-cells in the cutaneous microvasculature and migration into the epidermis remains poorly defined. After the T lymphocytes leave the bone marrow and enter the blood vessels, interaction between CLA on T lymphocyte and E-selectin on the dermal postcapillary venules leads to deposition of T lymphocytes in skin leading to formation of lesions of CTCL. Interestingly, in our case the patient had radiographic resolution of abdominal and lung nodes and a negative skin biopsy for MF. After myeloablative sUCB transplant, she also continued to have 100% donor chimerism in the bone marrow without any morphological or immunophenotypic evidence of lymphoma, yet she relapsed in the skin. This is most probably related to persistence of CTCL cells in the skin following ablative preparative regimen. The finding of mixed chimerism in the skin biopsy confirms that donor cells homed to cutaneous lymphoma sites; however, it appears to have failed in eliciting a GVL response in the skin.

This is one of the few cases reported that illustrates the differential response of extramedullary compared to intramedullary lymphoma to multiple standard combination chemotherapies and transplant, resulting in a relapse of CTCL skin lesions despite 100% donor chimerism at the bone marrow level. We also conclude that GVL conferred protection against systemic recurrence but failed to protect against CTCL skin recurrence. The pathologic mechanism for this differential response is poorly understood. We hypothesize that interplay of several mechanisms mediates this differential response including difference in microvascular supply. Chemotherapy and GVL effect may require bone marrow or lymph node microenvironment for better antilymphoma efficacy and hence explains its poor activity at skin lesions. Further investigation needs to be conducted to better understand the pathobiology of this differential response.

## Abbreviations

CTCL: Cutaneous T-cell lymphoma
WHO-EORTC: World Health Organization—European Organization for Research and Treatment of Cancer
MF: Mycosis fungoides
SS: Sézary syndrome
GVL: Graft-versus-lymphoma
sUCB: Single umbilical cord blood
UVB: Ultraviolet-B
PET: Positron emission tomography
CHOP: Cyclophosphamide, adriamycin, vincristine, and prednisone
ICE: Ifosfamide, carboplatin, and etoposide
cGy: Centigray
GvHD: Graft-versus-host disease
CD: Cluster of differentiation
CLA: Cutaneous lymphocytes associated antigen
CCR: Coreceptors
CCL: Chemokine (C-C motif) ligand.
